# Endogenous Anti-Inflammatory Very-Long-Chain Dicarboxylic Acids: Potential Chemopreventive Lipids †

**DOI:** 10.3390/metabo8040076

**Published:** 2018-11-03

**Authors:** Paul L. Wood

**Affiliations:** Metabolomics Unit, College of Veterinary Medicine, Lincoln Memorial University, 6965 Cumberland Gap Pkwy, Harrogate TN 37752, UK; paul.wood@lmunet.edu; Tel.: +1-865-585-1265

**Keywords:** cancer, very-long-chain dicarboxylic acids, anti-inflammatory, chemoprevention

## Abstract

In a paradigm shift, cancer research efforts are being dedicated to the discovery of chemopreventive agents. The goal of this approach is to delay or prevent the progression of augmented cell division to established cancer. Research has focused on dietary supplements, drugs, and endogenous lipids that possess anti-inflammatory properties. We undertook a lipidomics analysis of potential endogenous anti-inflammatory/anti-proliferative lipids in human plasma. We performed high-resolution mass spectrometric lipidomics analyses of plasma samples from controls and patients with colorectal, kidney, pancreatic, glioblastoma, and breast cancers. We present evidence that endogenous very-long-chain dicarboxylic acids (VLCDCA) are anti-inflammatory lipids that possess chemopreventative properties. In a family of VLCDCAs, we characterized VLCDCA 28:4, which is decreased in the plasma of patients with colorectal, kidney, and pancreatic cancers. The structure of this biomarker was validated by derivatization strategies, synthesis of the analytical standard, and tandem mass spectrometry. Our data suggest that VLCDCA 28:4 may be a useful blood biomarker for a number of cancers and that resupplying this lipid, via a prodrug for example, may offer a new anti-inflammatory therapeutic strategy for delaying or preventing the progression of cancer and other inflammatory diseases.

## 1. Introduction

Deep organ cancers such as colorectal (CRC), kidney, and pancreatic cancers are devastating malignancies which can only be detected by colonoscopy (CRC) or imaging [1,2,3,4]. Of significance, these cancers are characterized by extensive inflammation, which contributes to the tumor microenvironment and is associated with increased risk of developing cancer [5,6,7,8,9,10,11]. In this regard, we have found that decreased serum levels of very-long-chain dicarboxylic acids (VLCDCA), which are endogenous anti-inflammatory lipids, may be useful biomarkers of risk for kidney, colorectal, and pancreatic deep organ cancers.

Prior research of dietary [12] and pharmaceutical [13] anti-inflammatory/antioxidant agents has demonstrated varying degrees of potential as chemopreventive strategies. In addition, a number of lipids have been speculated to function as endogenous cancer chemoprevention systems. These include omega-3 polyunsaturated fatty acids [14], alpha-hydroxy-stearic acid [15], and 9-hydroxy-stearic acid [16]. Additionally, a family of lipids containing 28 to 36 carbons (444 to 555 amu), possessing anti-inflammatory and anti-proliferative properties in tissue culture [17], has been found to be decreased in the plasma of patients with CRC [18,19,20] and pancreatic cancers [21,22]. In a study of 4923 colonoscopy subjects, low serum levels of GTA/CRC-446 (gastrointestinal tract acid/colorectal cancer-446; C_28_H_46_O_4_, exact mass = 446.3396) correlated with a significantly increased risk for CRC [20]. In patients with CRC, the levels of GTA/CRC-446 were not restored to normal after surgery, but remained depressed, suggesting that they are not of tumor origin [18], consistent with stable steady-state plasma levels of GTA/CRC-446 monitored over a 1-year observation period in control subjects with no age-associated decrease in levels [18]. In toto, these data implicate that this family of anti-inflammatory lipids are potential endogenous chemopreventive agents. With regard to roles in controlling chronic inflammation, these lipids have also been found to be increased in the plasma of relapsing remitting multiple sclerosis patients, but decreased in secondary progressive multiple sclerosis patients [23].

The structures of this family of lipids have not been elucidated. The lipids were first misassigned as vitamin E metabolites [24], and subsequently, as hydroxylated, polyunsaturated ultra-long-chain fatty acids [18,19,20,21,22,23]. However, none of the conjectured lipid candidates were synthesized as analytical standards to validate the structural assumptions. We have characterized the family of 28 carbon lipids using high-resolution mass spectrometry (HR-MS) along with derivatization strategies and determined that they are not hydroxylated, polyunsaturated ultra-long-chain fatty acids, but are 28 carbon VLCDCAs with between 1 to 4 double bonds.

## 2. Results

### 2.1. HR-MS Analyses of C_28_H_46_O_4_

We focused our efforts on the full characterization of C_28_H_46_O_4_, since the mass of this previously misidentified lipid was most consistently decreased in the plasma of pancreatic and colorectal cancer patients [18,19,20,21,22]. Our preliminary HR-MS analyses of human plasma suggested that C_28_H_46_O_4_ could be a member of a family of 28 carbon VLCDCAs (Table 1). The tentative structure of VLCDCA 28:4n6 (C_28_H_46_O_4_) was:

**HOOC-(CH_2_)_4_-CH=CH-CH_2_-CH=CH-CH_2_-CH=CH-CH_2_-CH=CH-(CH_2_)_11_-COOH**

This tentative structure was based on our validation that the lipid was a dicarboxylic acid and that there is only one published very-long-chain-fatty acid (VLCFA) that could serve as a direct precursor of VLCDCA 28:4. This direct precursor of VLCDCA 28:4n6 would be VLCFA 28:4n6, which has previously been identified (VLCFA 28:4n6; C_28_H_48_O_2_; LMFA01030825) as a free fatty acid and as a fatty acid substituent in phosphatidylcholines and sphingomyelins in ocular tissue, sperm, testes, and brain [25,26,27]:

**H_3_C-(CH_2_)_4_-CH=CH-CH_2_-CH=CH-CH_2_-CH=CH-CH_2_-CH=CH-(CH_2_)_11_-COOH**

### 2.2. Structural Validation of VLCDCA 28:4

To validate the proposed dicarboxylic structure of VLCDCA 28:4, we used picolylamine, which derivatizes carboxylic functions. The dipicolinyl derivative of VLCDCA 28:4 isolated from 3 mL of human plasma, semipurified by basic anion exchange, was generated. The molecular cation of dipicolinyl-VLCDCA 28:4 at [*m*/*z* 627.4632]^+^ (446.3396 + 2 × 90.0582 = 626.4560) was monitored with 0.66 ppm mass error, definitively validating the hypothesis that this lipid is a dicarboxylic acid. The molecular cation [495.5138]^+^ of the dipicolinyl derivative of the internal standard [^2^H_28_]dicarboxylic acid 16:0 (314.3901 + 2 × 90.0582 = 494.5065) was observed with a mass error of 0.17 ppm and demonstrated >99% reaction completion. These data clearly validate the dicarboxylic structure of VLCDCA 28:4; however, the hypothesized positions of the double bonds were based on the assumption that previously reported VLCFA 28:4n6 could be the direct precursor.

To address this issue, we next synthesized the analytical standard for VLCDCA 28:4n6 and validated the double bond locations via MS^2^ analysis of the standard and a biological extract, semipurified by basic anion exchange. The dominant MS^2^ fragments for both the analytical standard and biological extract resulted from the loss of water and decarboxylation. Further analysis of the minor fragments observed with MS^2^ revealed the ω1 fragment (C_6_H_10_O_2_) and ω2 fragment (C_9_H_14_O_2_) validating the z22 and z19 double bonds, respectively. These fragments are common to both the synthetic standard and the endogenous biomolecule (Table 2). The α1 fragment (C_13_H_24_O_2_) and α2 fragment (C_16_H_28_O_2_) also validated the z13 and z16 double bonds, respectively (Table 2).

### 2.3. VLCDCA 28:4 in Biofluids

We monitored for VLCDCA 28:4 levels in a number of human biofluids and in plasma from Cynomologous monkeys, Rhesus macaque monkeys, dogs, rats, cows, and cats. In this regard, VLCDCA 28:4 was monitored in all human biofluids examined (Table 3), including adult plasma, umbilical cord plasma, synovial fluid, aqueous humor, pleural fluid, and cerebrospinal fluid. Of key interest are the observations that VLCDCA 28:4 was only found in human plasma and that of closely related primates, but not in the other species examined (Table 3). 

### 2.4. Plasma VLCDCA 28:4 in Cancer Patients

**Figure 1:**
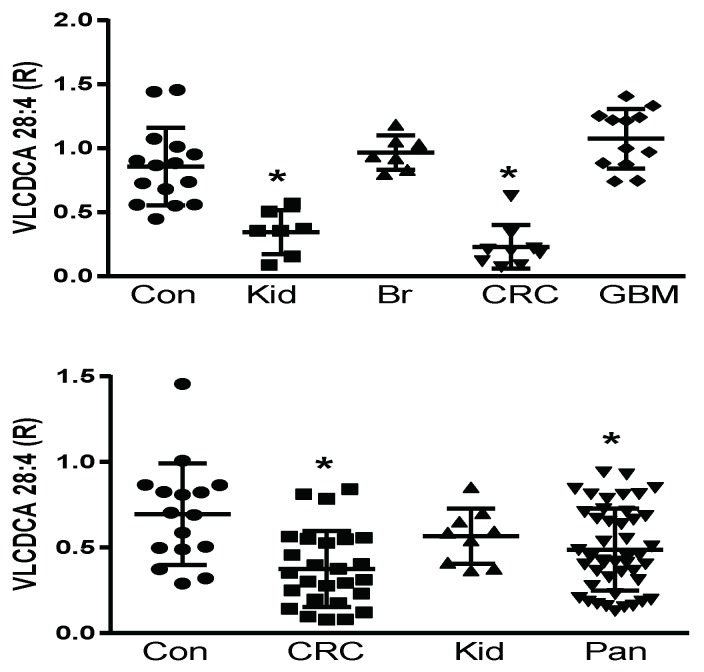
**Upper Figure:** Pilot study of plasma levels (100 μL) of VLCDCA 28:4 in controls (Con; *N* = 15) and patients with kidney (Kid; *N* = 7), breast (BR; *N* = 7), colorectal (CRC; *N* = 9), or glioblastoma multiforme (GBM; *N* = 12) cancers. **Lower Figure:** Validation study of plasma levels (100 μL) of VLCDCA 28:4 in controls (Con; *N* = 16) and patients with colorectal (CRC; N = 26), kidney (Kid; N = 9), or pancreatic (Pan; *N* = 45) cancers. R represents ratio of the peak area of endogenous VLCDCA 28:4 to the peak area of 1 nanomole of [^2^H_28_]DCA 16:0. *, *p* < 0.01 (*t*-test).

Prior to the structural elucidation of VLCDCA 28:4, by monitoring the anion of this lipid, it was found to be decreased in the plasma of colorectal and pancreatic cancer patients, but not to be altered in patients with breast, prostate, or liver cancers [18,19,20,21,22,24]. We undertook a pilot and validation study to further verify these observations in colorectal and pancreatic cancers and to further examine pilot populations of several other cancers, where blood samples were available commercially or from a collaborator. With these studies, we validated previous findings of decreased VLCDCA 28:4 levels in the plasma of patients with colorectal cancer and pancreatic cancer and extended these observations to detect decreased levels of VLCDCA 28:4 in kidney cancer patients. By contrast, no alterations in the circulating levels of VLCDCA 28:4 were monitored in patients with breast cancer or glioblastoma multiforme (Figure 1; upper figure). These data indicate that it is essential to next identify the molecular targets of VLCDCA 28:4 to understand the varying roles of this anti-inflammatory lipid in different forms of cancer. In addition, the patient numbers we studied are small and need to be increased with larger patient cohorts to monitor for patient heterogeneity within a given cancer diagnosis. We are currently conducting such a study with larger CRC patient cohorts from Italy and Brazil.

### 2.5. VLCDCA 28:4: Anti-Inflammatory Activity

**Figure 2:**
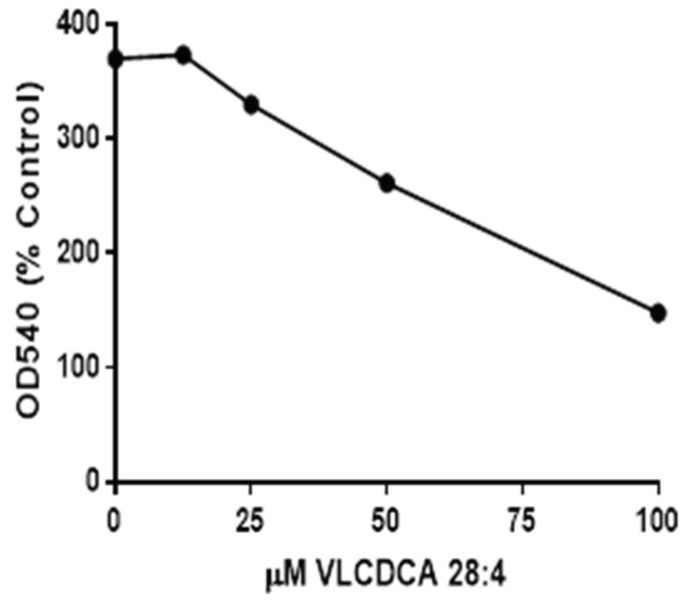
Inhibition, by VLCDCA 28:4, of lipopolysaccharide (LPS) (1 µg/mL of media) stimulation of nitric oxide (NO) release into the medium of THP-1 monocytes incubated for 48 h. in 24 well plates. NO was measured as nitrite in the medium using the Griess colorimetric procedure^40^. The 0 concentration point represents the NO production by LPS, while other points represent LPS and coincubation with VLCDCA 28:4 in ethanol. The control and LPS wells also received an equal volume of ethanol. Values are the mean values of 8 wells, with the RSD ranging from 3.8 to 10.2%.

Previous evaluations of semipurified extracts of the human plasma lipid fraction containing masses 440–560 demonstrated anti-inflammatory properties in tissue culture [17]. Our evaluations of synthetic VLCDCA 28:4 have shown that this dicarboxylic acid has anti-inflammatory activity in vitro, blocking the ability of lipopolysaccharide (LPS) to stimulate nitric oxide production in human monocytes (Figure 2).

## 3. Discussion

**Figure 3:**
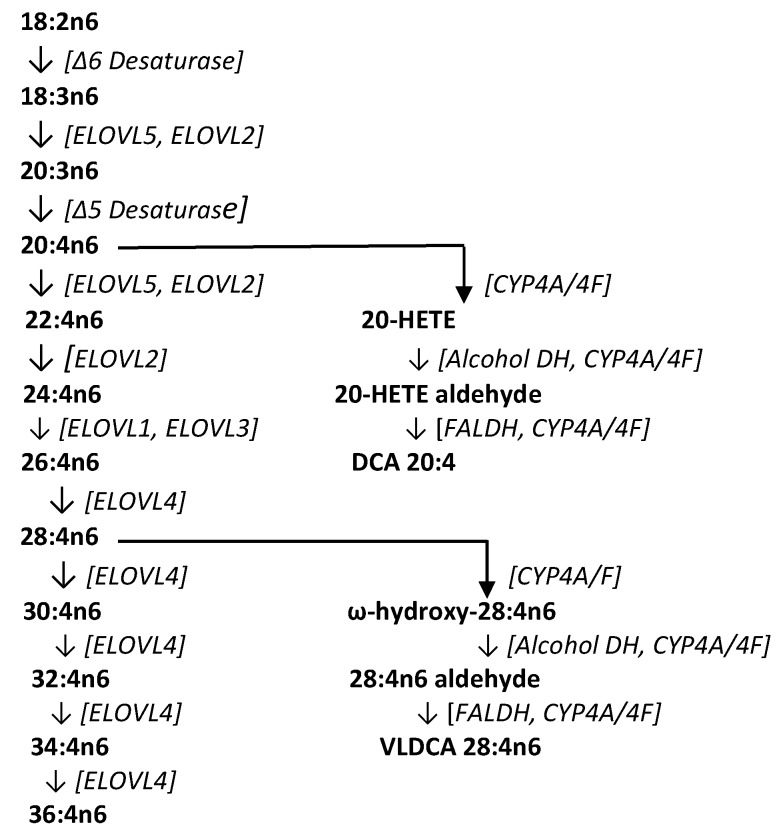
Biosynthetic pathway for very-long-chain fatty acids (VLCFA), the dicarboxylic acid 20:4 (DCA 20:4), and the very-long-chain dicarboxylic acid 28:4 (VLDCA 28:4). COX, cyclooxygenase; CYP, cytochrome P450; ELOVL, elongation of very-long-chain fatty acids; FALDH, fatty aldehyde dehydrogenase.

Previous research (Figure 3) has identified very-long-chain fatty acids (VLCFA) of up to 36 carbons formed by sequential fatty acid elongation with ELOVL4 (elongation of very-long-chain fatty acids-4), an enzyme found at moderate levels in the brain, spleen, pancreas, kidney, ileum, and lymph nodes, and high levels in the retina, thymus, epidermis, and sperm [25,26,27]. These VLCFAs perform structural functions as fatty acid components of sphingomyelins and phosphatidylcholines, serve signal transduction roles, and are potential precursors to dicarboxylic acids [27,28]. Conversion of VLCFAs to dicarboxylic acids first involves ω-hydroxylation of the fatty acid by microsomal CYP4A/4F enzyme systems (CYP4A11, CYP4F2, CYP4F3A, CYP4F3B) [28,29,30,31]. The CYP4 gene family constitutes more than 63 individual members with specific substrate affinities and unique regional tissue distributions. One of the functions of these enzymes is the inactivation of inflammatory lipids, while another involves the metabolism of VLCFAs to their ω-hydroxy derivatives [28,29,30,31]. An example of this involves the conversion of 20-HETE to ω-hydroxy arachidonic acid, and the subsequent conversion to 20-carboxy arachidonic acid (DCA 20:4) by alcohol dehydrogenase and fatty aldehyde dehydrogenase [32]. In CRC tissues, the generation of VLCDCAs is presumably curtailed as a result of both decreased CYP4F expression [33] and hypermethylation of ELOVL4 [34].

Of relevance to our VLCDCA clinical data are the observations that acetylsalicylic acid [35] and statins [13] are inducers of CYP4 enzymes. These drugs both significantly reduce the risk for pancreatic and colorectal cancers [12,13,35]. It is interesting to speculate that the chemopreventive actions of these drugs may involve augmentation of the biosynthesis of VLCDCAs catalyzed by CYP4 [28,31]. 

The roles of alcohol and aldehyde dehydrogenases in colorectal cancer are not clear at this time, with multiple isoforms and differential protein expression to be considered. Primates have retained alcohol dehydrogenases 1 to 5 (ADH1-5), but lost ADH6 [36]. While ADH1-4 are highly conserved, ADH5 is very divergent [36], and therefore an isoform of ADH5 may be involved in VLCDCA synthesis in humans. Evaluation of the role of aldehyde dehydrogenases (ALDH) in the biosynthesis of VLCDCAs is complicated in that there are 19 isozymes in this family [37]. There are also a number of ALDH polymorphisms that are currently being studied in kidney and colorectal cancers [38]. The potential role of aldehyde oxidase 1 (AOX1) in the synthesis of VLCDCAs also remains to be investigated.

## 4. Materials and Methods 

### 4.1. Clinical Samples

Depersonalized plasma samples and biofluids were purchased from Innovative Research, BioChemed Services, and the Cooperative Human Tissue Network (CHTN). Depersonalized glioblastoma multiforme plasma samples were supplied by Dr. Charles Conrad, MD Anderson Cancer Center (ClinTrials #NCT00805376). Patient demographics are presented in Table 4.

### 4.2. Sample Processing

One hundred microliters of plasma were mixed with 1 mL of water and 1 mL of methanol containing [^2^H_28_]dicarboxylic acid 16:0 as the internal standard [39,40]. The tubes were vigorously shaken at room temperature for 30 min after the addition of 2 mL of methyl-tert-butyl ether (MTBE). After centrifugation at 4000× *g* for 30 min, 1 mL of the upper organic layer was dried by centrifugal vacuum evaporation and dissolved in 150 μL of isopropanol:methanol:chloroform (4:2:1) containing 15 mM ammonium acetate. Extraction efficiency was 98 to 99%.

### 4.3. Semipurification of Plasma VLCDCAs

For structural validation studies, VLCDCAs were semipurified from MTBE–ethanol extracts of 3 or 5 mL of human plasma via a basic anion exchanger (HyperSep Sax, Thermo Fisher, Waltham, MA, USA). The column was conditioned by the sequential addition of 2 mL of methanol, 2 mL of water, and 2 mL of acetonitrile. The methanol and methyl–tert–butylether extract of human plasma was dried by vacuum centrifugation and re-dissolved in 2 mL of methanol which was applied to the conditioned column. The column was washed by the sequential addition of 2 mL of water, 2 mL of acetonitrile, and 2 mL of methanol. The dicarboxylic acids were then eluted with 2 mL of acetonitrile:methanol:formic acid (50:50:3). This eluate was dried by vacuum centrifugation and dissolved in acetonitrile:methanol (1:1) for mass spectrometric analysis or used for derivatization reactions. 

### 4.4. Chemical Synthesis of VLCDCA 28:4

Using a published synthetic scheme [41], ω-hydroxy-20:4n6-methyl ester (Compound 10) was obtained, locking the required double bond positions relative to the ω-terminal of the target dicarboxylic acid. The subsequent reactions are outlined below:

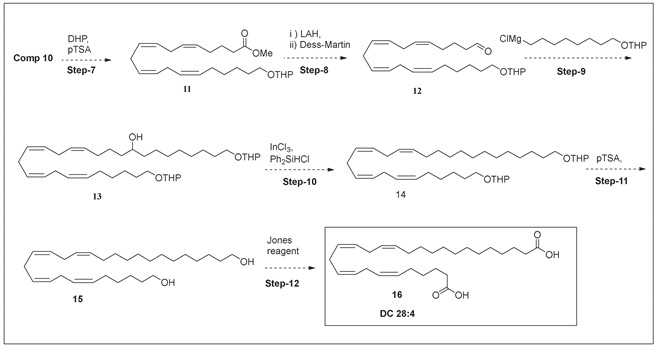



The final product was validated by NMR (Supplementary Material) and by HR-MS. HPLC–MS demonstrated 99.90 % purity, and MS^2^ (Table 2) validated the double bond locations. With regard to the NMR, the protons due to carboxylic acid were seen as a singlet at 11.96 ppm. The olefin protons (6, 7, 9, 10, 12, 13, 15, 16) could be seen as broad multiplets between 5.20–5.40 ppm. The homoallylic protons (8, 11, 14), were seen as broad multiplets between 2.75–2.85 ppm, while the allylic protons (5, 17) appeared as broad multiplets between 1.90–2.10 ppm. The protons adjacent to the carboxylic acid functional group (2, 27) appeared as multiplets between 2.10–2.20 ppm. The rest of the aliphatic protons appeared as multiplets between 1.20–1.60 ppm.

### 4.5. High-Resolution Mass Spectrometric Analyses

For all analyses, samples underwent direct infusion analyses at a flow rate of 12 μL per min. Samples were analyzed via high-resolution mass spectrometry (HR-MS) using a Q-Exactive benchtop orbitrap (Thermo Fisher) with a resolution of 140,000 and less than 3 ppm mass error. Electrospray ionization (ESI) with a sheath gas of 12, a spray voltage of 3.7 kV, and a capillary temperature of 321 °C were used. For MS^2^ studies, a window of 1.5 amu was used for the precursor ion and the product ions were obtained at high resolution (<3 ppm mass error). For MS^2^ studies, the neutral collision energy (NCE) was optimized between 20 and 30 eV.

### 4.6. Derivatization: Picolylamine

Validation of 2 carboxylic groups in VLCDCA 28:4 was obtained by derivatization with 2-picolylamine [42]. Briefly, to 3 mL of dried plasma lipid extracts, purified by anion exchange, were added 20 μL of triphenylphosphine (2.6 mg/mL acetonitrile), 20 μL dithiopyridine (2.2 mg/mL acetonitrile), and 20 μL 2-picolylamine (20 μL 2-picolylamine/ml acetonitrile). The samples were heated with shaking at 60 °C for 10 min and then dried by vacuum centrifugation prior to dissolution in acetonitrile:methanol (1:1). In positive ion ESI, the cations of the dipicolinyl derivatives of dicarboxylic acids were quantitated (addition of 90.058183 per carboxylic functional group). 

The tandem mass spectrum of di–PA–VLCDCA 28:4 (C_40_H_58_N_4_O_2_) was dominated by *m*/*z* 109.0760 (base peak), the molecular cation for picolylamine (PA, 1.9 ppm mass error). The next most prominent ion was 519.3945 (C_34_H_50_N_2_O_2_; [M + H − PA]^+^; 2.3 ppm mass error), followed by 501.3839 (C_34_H_48_N_2_O_1_; [M + H − PA − H_2_O]^+^; 1.7 ppm mass error). The tandem [M + H − PA − H_2_O]^+^ ion has the potential to be very useful for the development of a clinical assay using tandem mass spectrometry, since this product ion is highly specific and can be monitored with high mass accuracy by either tandem quadrupole-orbitrap or quadrupole-time-of-fight analysis. 

### 4.7. Anti-Inflammatory Assay

THP-1 (human macrophage) cells (ATTC) were cultured in 24-well plates in RPMI media containing 10% FBS and Pen-Step until 90% confluent. Wells were next treated with lipopolysaccharide (Sigma L45116; 1 μg/mL of fresh medium). Varying concentrations of synthetic VLCDCA 28:4 were added to the wells in ethanol and the plates were incubated for 48 h. The control wells and LPS-alone wells also received ethanol without VLCDCA 28:4. After 48 h of incubation, 100 μL of media from each well was transferred to a 96-well microplate. To these wells were added 100 μL of Griess reagent and the plate was read at 540 nm after 14 min to quantitate nitrite released into the medium after induction of nitric oxide synthase by LPS [43].

## 5. Conclusions

In summary, there are a number of bioactive lipids that are involved in inflammation and cell proliferation. Similarly, there are a number of anti-inflammatory lipid mediators that include lipoxins derived from *n*-6 arachidonic acid and resolvins, protectins, and maresins derived from *n*-3 polyunsaturated fatty acids. Of particular interest, the anti-inflammatory actions of lipoxin A4 have been shown to block the differentiation of pancreatic tumor stroma [44]. All of these lipid pro-resolving mediators are synthesized on demand to resolve ongoing inflammatory responses and are not stored or maintained at a steady-state level. In contrast, anti-inflammatory VLCDCAs appear to be maintained at a steady-state plasma level, with decreases in this steady-state increasing the risk of kidney, colorectal, and/or pancreatic cancer development.

In conclusion, our data suggest that VLCDCAs represent a new class of anti-inflammatory lipids. In the case of VLCDCA 28:4, this may represent a useful biomarker for deep organ cancer risk. Resupply of VLCDCAs may also offer a new therapeutic approach for cancer chemoprevention and/or treatment. Prodrugs of endogenous VLCDCAs represent one such approach to rapidly evaluate this opportunity. In addition, it is essential to understand the roles of CYP4A/4F, alcohol dehydrogenase, and aldehyde dehydrogenase in the generation of human VLCDCAs for the maintenance of steady-state levels that are both anti-inflammatory and chemopreventive in biofluids.

## 6. Patents

Identification and use of very-long-chain dicarboxylic acids for disease diagnosis, chemoprevention, and treatment. (Paul L. Wood) USPTO 15/284,219

## Figures and Tables

**Table 1:** High-resolution mass spectrometry (HR-MS) analyses of human plasma 28 carbon very-long-chain dicarboxylic acids (VLCDCAs). The molecular anions of endogenous VLCDCAs were monitored in organic extracts of 0.1 mL of human plasma. R, ratio of the peak area of the endogenous VLCDCA to the peak area of 1 nanomole of the internal standard [^2^H_28_]dicarboxylic acid (DCA)16:0.

VLCDCA	Molecular Formula	Published Nomenclature [17,18,19,20,21,22]	Exact Mass	[M − H]^−^	R	ppm Mass Error
[^2^H_28_] DCA 16:0	Internal std.		314.39016	313.3829		1.60
28:0	C_28_H_54_O_4_		454.4022	453.3949	0.067	1.56
28:1	C_28_H_52_O_4_	GTA-452	452.3866	451.3793	0.276	1.30
28:2	C_28_H_50_O_4_	GTA-450	450.3709	449.3636	0.773	1.73
28:3	C_28_H_48_O_4_	GTA-448	448.3553	447.3480	1.084	1.24
28:4	C_28_H_46_O_4_	GTA-446	446.3396	445.3323	0.778	1.18
28:5	C_28_H_44_O_4_		444.3240	443.3167	0.047	0.22

**Table 2:** HR-MS and MS^2^ of the synthetic standard of VLCDCA 28:4 and VLCDCA 28:4 extracted from 3 mL of human plasma and semipurified by anion exchange chromatography. Collision energy of 25. ppm Std., ppm mass error for the synthetic standard; ppm Extract, ppm mass error for the human plasma lipid extract.

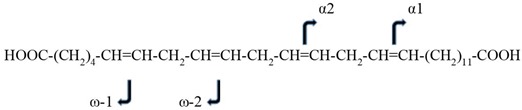				
Fragment	Formula	Calculated Anion	ppm Std.	ppm Extract
VLCDCA 28:4	C_28_H_46_O_4_	445.3323	0.62	1.07
H_2_O Loss	C_28_H_44_O_3_	427.3217	0.39	0.30
CO_2_ and H_2_O Loss	C_27_H_44_O	383.3319	0.05	0.44
CO_2_ Loss	C_27_H_46_O_2_	401.3425	0.07	0.36
ω-1	C_6_H_10_O_2_	113.0608	0.09	0.14
ω-2	C_9_H_14_O_2_	153.0921	0.07	0.30
ω-2-H_2_O	C_9_H_12_O	135.0815	0.14	0.89
α-1	C_13_H_24_O_2_	211.1703	0.14	0.66
α-1-H_2_O	C_13_H_22_O	193.1597	0.31	0.92
α-2	C_16_H_28_O_2_	251.2016	0.34	0.48
α-2-H_2_O	C_16_H_26_O	233.1910	0.13	0.13

**Table 3:** VLCDCA 28:4 levels in the plasma of different species and in different human biofluids. VLCDCA 28:4 levels are presented as R (ratio of endogenous VLCDCA 28:4 peak area to the peak area of 1 nanomole [^2^H_28_]dicarboxylic acid 16:0 per 100 μL biofluid). In the cases where VLCDCA 28:4 was not detected in 100 μL biofluid, the negative observations were further validated by extracting 500 μL biofluid. The values are the means of 3 observations. ND, not detected.

Biofluid	R (N)
Adult human plasma	0.79 (12)
Human synovial fluid	0.50 (3)
Human pleural fluid	0.14 (3)
Human cerebrospinal fluid	0.045 (12)
Human umbilical cord plasma	0.85 (6)
Human aqueous humor	0.024 (2)
Chimpanzee plasma	0.061 (2)
Cynomologous monkey plasma	0.0022 (2)
Rhesus macaque plasma	ND (1)
Dog plasma	ND (6)
Rat plasma	ND (6)
Cow plasma	ND (6)
Cat plasma	ND (6)

**Table 4:** Patient demographics for plasma VLCDCA 28:4 measurements. All patients included in the study were Caucasian.

Group	Age (Yr. ±SD)	N	Number of Females
Pilot-Controls	54.5 ± 8.9	15	9
Pilot-Kidney	65.8 ± 12.0	7	1
Pilot-Breast	59.4 ± 7.4	7	7
Pilot-GBM	54.6 ± 5.6	12	5
Pilot-CRC	69.9 ± 6.9	9	5
Validation-Controls	57.3 ±5.8	23	14
Validation-Pancreatic	69.1 ± 9.3	18	11
Validation-CRC	57.3 ± 8.1	28	22
Validation-Kidney	55.7 ± 8.9	9	2

## Supplementary Materials

Available online at http://www.mdpi.com/2218-1989/8/4/76/s1.
