# Supplementary material for: Endogenous Anti-Inflammatory Very-Long-Chain Dicarboxylic Acids: Potential Chemopreventive Lipids
Source: Metabolites. 2018 Nov 3;8(4):76. doi: 10.3390/metabo8040076 (PMC6315409; doi:10.3390/metabo8040076)
Supplement: Supplementary file 1 [file metabolites-08-00076-s001.pdf]

Sample Name:
E17063-41-02
Data Collected on:
Sai_vnmrs400-vnmrs400
Archive directory:
/home/vnmr1/data/2017/Jul/20170710
Sample directory:
E17063-41-02_20170710_01
FidFile: E17063-41-02_PROTON_01

Plotting Time: Mon Jul 10 10:49:20 IST 2017
Pulse Sequence: PROTON (s2pul)
Solvent: dmso
Data collected on: Jul 10 2017

Temp. 22.3 C / 295.4 K
Operator: vnmr1

Relax. delay 2.000 sec
Pulse 30.0 degrees
Acq. time 2.045 sec
Width 8012.8 Hz
8 repetitions
OBSERVE H1, 399.9662042 MHz
DATA PROCESSING
FT size 32768
Total time 1 hr, 9 min

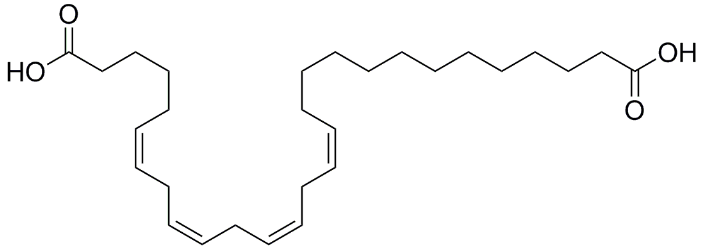

DC 28:4 (M.W 446)

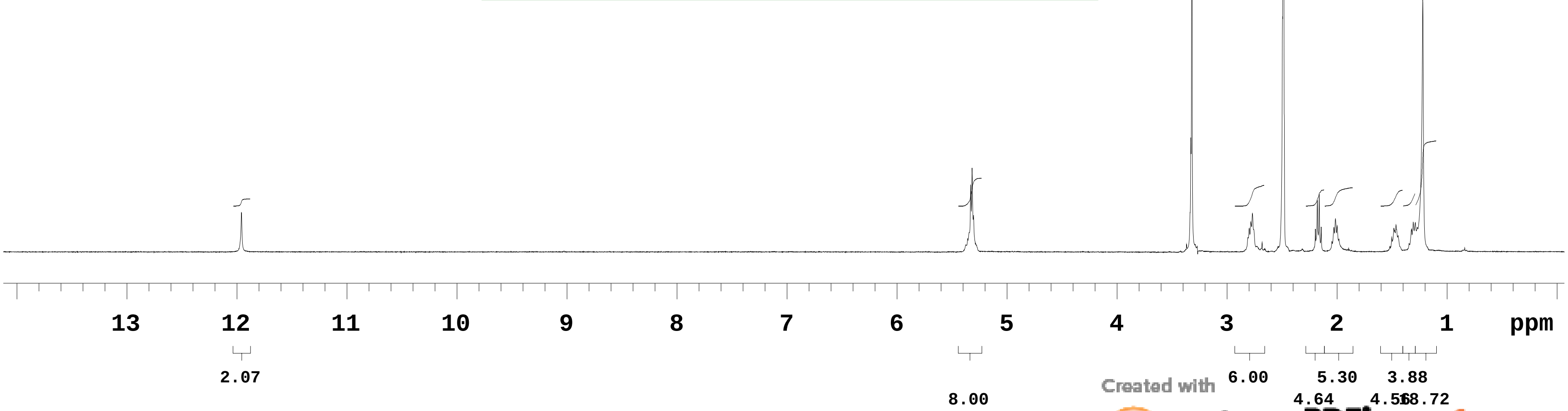

Plotname: E17063-41-02_PROTON_01_plot01

Created with
nitroPDF professional
download the free trial online at nitropdf.com/professional

Sample Name:
E17063-41-02
Data Collected on:
Sai_vnmrs400-vnmrs400
Archive directory:
/home/vnmr1/data/2017/Jul/20170710
Sample directory:
E17063-41-02_20170710_01
FidFile: E17063-41-02_PROTON_01

Pulse Sequence: PROTON (s2pul)
Solvent: dmso
Data collected on: Jul 10 2017

Temp. 22.3 C / 295.4 K
Operator: vnmr1

Relax. delay 2.000 sec
Pulse 30.0 degrees
Acq. time 2.045 sec
Width 8012.8 Hz
12 repetitions
OBSERVE H1, 399.9662042 MHz
DATA PROCESSING
FT size 32768
Total time 1 hr, 9 min

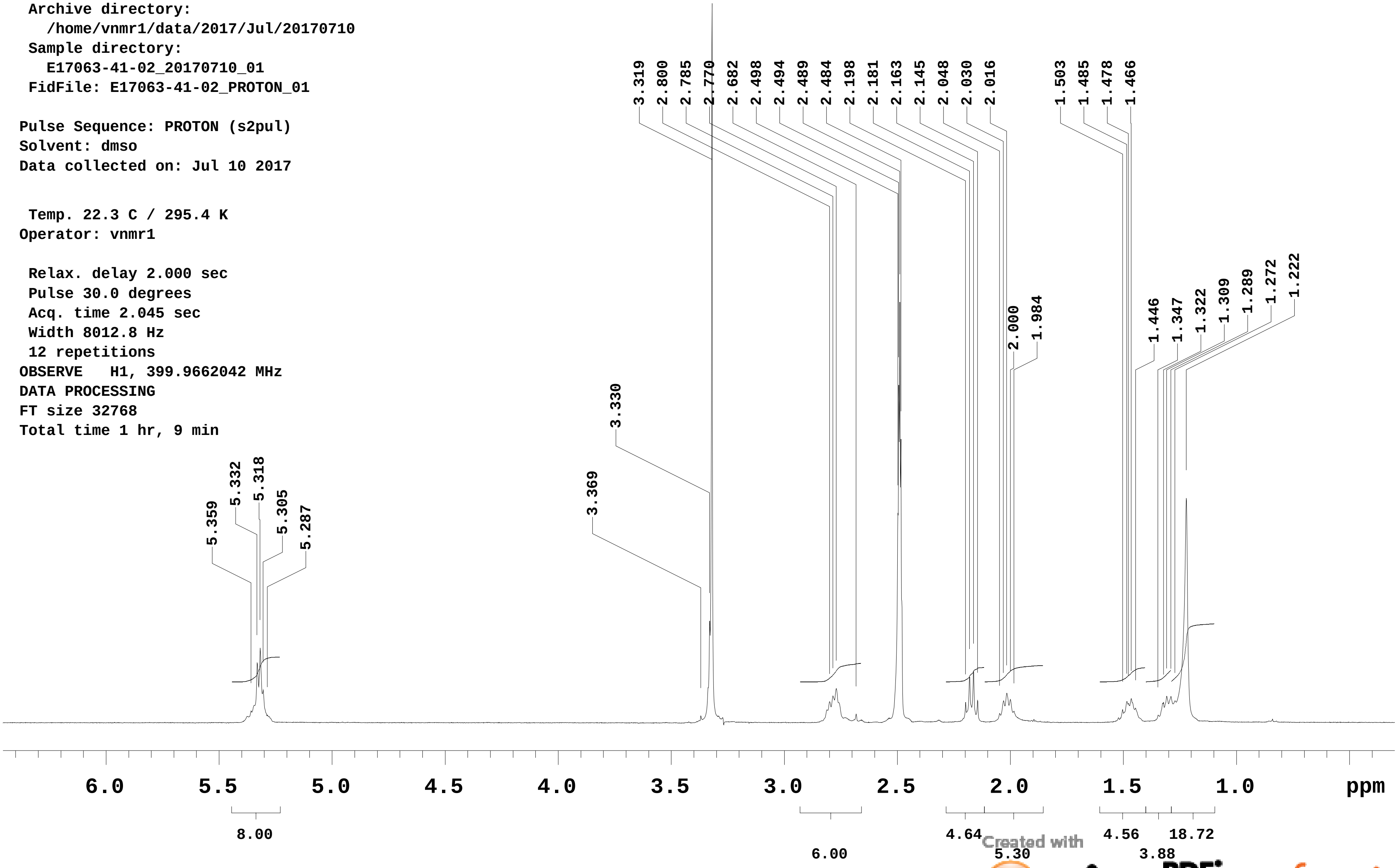

Plotname: E17063-41-02_PROTON_01_plot03

Created with nitroPDF professional
download the free trial online at nitropdf.com/professional

Sample Name:
E17063-41-02
Data Collected on:
Sai_vnmrs400-vnmrs400
Archive directory:
/home/vnmr1/data/2017/Jul/20170710
Sample directory:
E17063-41-02_20170710_01
FidFile: E17063-41-02_PROTON_01

Pulse Sequence: PROTON (s2pul)
Solvent: dmso
Data collected on: Jul 10 2017

Temp. 22.3 C / 295.4 K
Operator: vnmr1

Relax. delay 2.000 sec
Pulse 30.0 degrees
Acq. time 2.045 sec
Width 8012.8 Hz
12 repetitions
OBSERVE H1, 399.9662042 MHz
DATA PROCESSING
FT size 32768
Total time 1 hr, 9 min

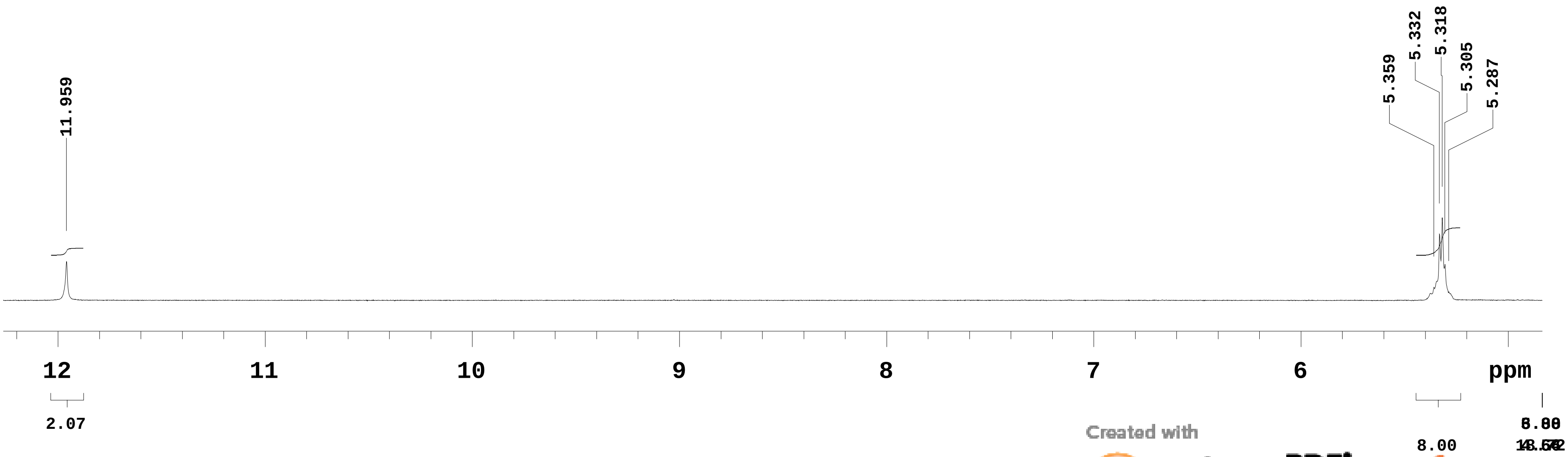

Plotname: E17063-41-02_PROTON_01_plot02

Created with
nitroPDF professional
download the free trial online at nitropdf.com/professional

Sample Name:
E17063-041-02_NMR
Data Collected on:
Sai_vnmrs400-vnmrs400
Archive directory:
/home/vnmr1/data/2017/Jul/20170707
Sample directory:
E17063-041-02_NMR_20170707_01
FidFile: E17063-041-02_NMR_PROTON_01

Plotting Time: Fri Jul 7 15:44:07 IST 2017
Pulse Sequence: PROTON (s2pul)
Solvent: cdcl3
Data collected on: Jul 7 2017

Temp. 25.0 C / 298.1 K
Operator: vnmr1

Relax. delay 2.000 sec
Pulse 30.0 degrees
Acq. time 2.000 sec
Width 8012.8 Hz
8 repetitions
OBSERVE H1, 399.9643004 MHz
DATA PROCESSING
FT size 32768
Total time 1 hr, 8 min

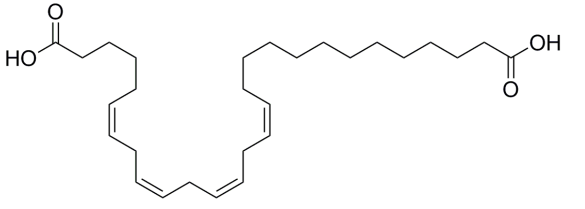

DC 28:4 (M.W 446)

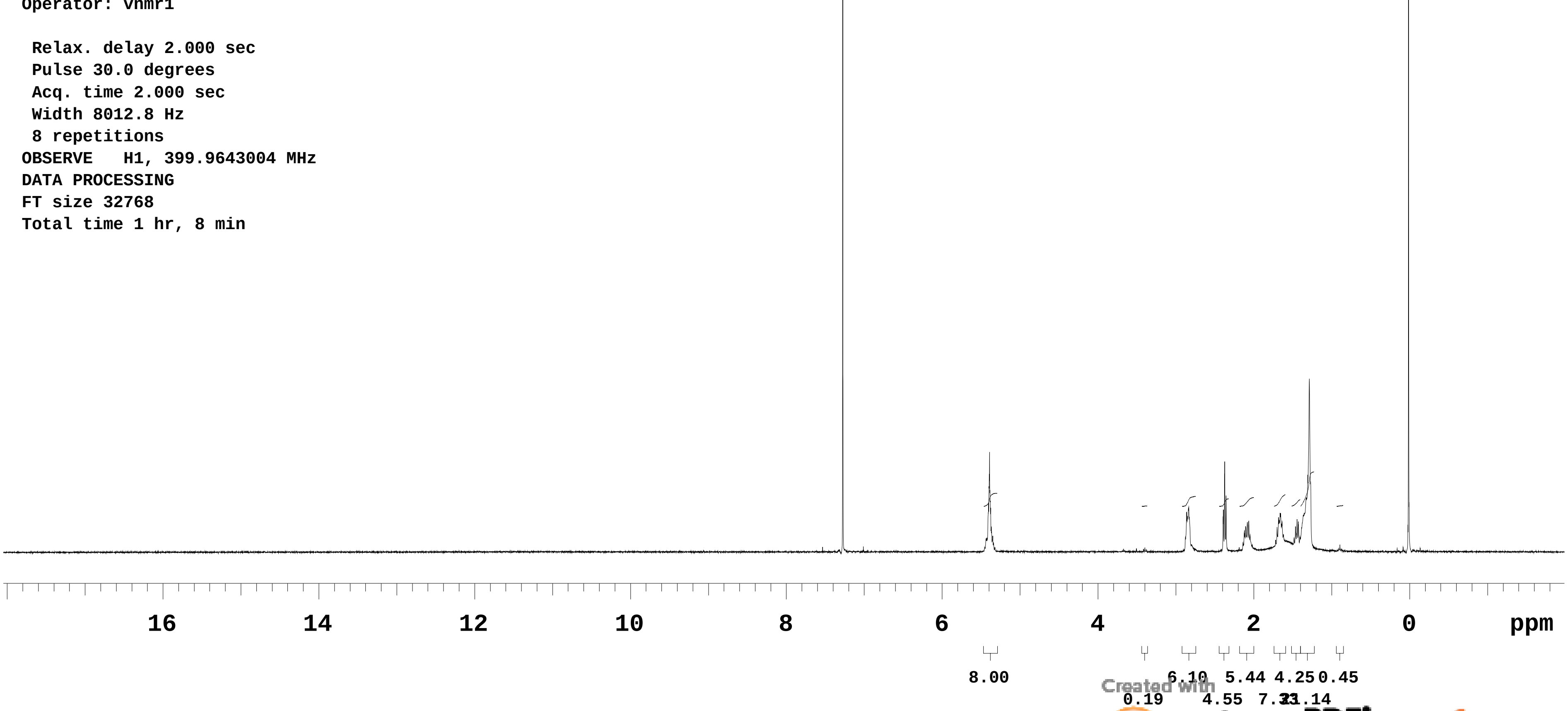

Plotname: E17063-041-02_NMR_PROTON_01_plot01

Created with
nitroPDF professional
download the free trial online at nitropdf.com/professional
